# Drinking Water Availability in Public Schools: An Assessment of Four New Jersey School Districts

**DOI:** 10.3390/ijerph22091332

**Published:** 2025-08-27

**Authors:** Cong Wang, Francesco Acciai, Sarah Martinelli, Punam Ohri-Vachaspati

**Affiliations:** 1 College of Medicine, University of Arizona, Phoenix, AZ 85004, USA; crw5@arizona.edu; 2 College of Health Solutions, Arizona State University, 550 North 3rd Street, Phoenix, AZ 85004, USA; facciai@asu.edu (F.A.); punam.ohri-vachaspati@asu.edu (P.O.-V.)

**Keywords:** school drinking water availability, Healthy, Hunger-Free Kids Act and water availability, water availability in schools

## Abstract

Background: The Healthy, Hunger-Free Kids Act requires access to free drinking water in public school cafeterias during meal times. Previous studies suggest that increasing access to water in schools can increase its consumption among students, potentially reducing their intake of sugar-sweetened beverages. The current study provides a descriptive assessment of water availability, delivery methods, and quality in 96 public schools across four New Jersey school districts. Methods: As part of the New Jersey Child Health Study, we administered an online and paper survey to school nurses at 96 schools to evaluate the availability, delivery, and quality of water in the school cafeteria during lunchtime. Univariate and bivariate statistics were used to analyze the data. Results: In school year 2019–20, 83 (86.5%) schools reported providing free drinking water in the cafeteria during lunch. The most common source of water was water fountains, available in 64 schools (66.7%). Issues related to water quality, specifically cleanliness, temperature, pressure, and taste, were present in 20–30% of schools. Conclusions: While most schools surveyed provide access to clean, free drinking water, students in some schools still lack free access to drinking water during school meals, which may affect overall water consumption among students.

## 1. Introduction

As of 2020, approximately 14.7 million children aged 2–19 years had obesity (defined as having a Body Mass Index or BMI greater than the 95th percentile for age and sex) in the US [1]. Rates of childhood obesity are disproportionately higher among children in families with lower incomes and among Hispanic children and Non-Hispanic Black children compared to their white counterparts [1]. Childhood obesity is associated with adverse mental health outcomes, increased likelihood of elevated weight in adulthood, and early onset of conditions such as elevated blood glucose, hypertension, and fatty liver [2,3]. The development of obesity is a complex process that includes interactions between individual and environmental factors. One important contributor is the overconsumption of energy-dense, sugar-sweetened beverages (SSBs) [4,5,6,7], which are the leading source of added sugar in the American diet. A recent meta-analysis highlighted that in adolescents, each additional serving of SSB was linked to a 0.07 kg/m^2^ higher BMI [7]. Randomized controlled trials further supported this association, showing that reducing SSB intake led to smaller BMI gains compared to controls [7]. Drinking water, on the other hand, is associated with benefits beyond weight status, such as physical and cognitive functioning, including improvements in concentration, alertness, and short-term memory, and, when fortified with fluoride, is associated with fewer dental caries [8,9]. A recent study found that consumption of water in elementary school children was impacted by water availability more so than the active promotion of water consumption [10]. This suggests that providing access to water in schools could increase its consumption among students even without ad hoc water promotion campaigns. Further, since there is often an inverse correlation between water consumption and SSB intake [11], increasing water availability in schools is a potential strategic intervention to address rising obesity rates in children [12].

The Healthy, Hunger-Free Kids Act (HHFKA) of 2010 (USDA PL 111-296) included provisions to promote healthier beverage consumption among school children during the school day [13]. The legislation set nutrition guidelines for beverages served in school meals and sold a la carte and mandated the availability of free drinking water in cafeterias during meals. However, the language in HHFKA did not specify the mode of water access, nor did it provide any language about the number or type of water sources required, which may limit the reach of the policy. Students’ concerns about the cleanliness and safety of certain sources of drinking water may also limit their intake [14,15], suggesting that both the availability and quality of drinking water must be considered. Several studies, mostly conducted prior to or just after HHFKA implementation, reported on the availability, delivery methods, and quality of water in schools [16,17,18,19,20]. Prior to the HHFKA, less than half of California schools provided water in the cafeteria at lunch time [20], and nationally, only 70% of high schools reported having access to free water during lunch during the 2011–2012 school year (SY) [18]. Studies conducted just after HHFKA implementation found that there were increases in water availability and that water availability was associated with greater water intake [17]. However, some schools still struggled to meet students’ needs in terms of providing access to water that is appealing to students [16,19,21], in part due to a lack of infrastructure and students’ distrust of the quality of available tap water. 

Despite the national policy mandate in HHFKA to provide water in school cafeterias, there is limited data on the availability and quality of water sources in schools. This study addresses that gap by assessing water access nearly a decade after HHFKA implementation in four New Jersey districts where obesity rates remain a public health concern, rising from 25% in SY 2013–14 to 29% in SY 2019–20 [22].

## 2. Materials and Methods

This study is a secondary data analysis of a larger, longitudinal study, the New Jersey Childhood Health Study, which examined the impact of school and neighborhood environments on childhood obesity rates. The study was conducted in four low-income cities in New Jersey—Camden, Newark, New Brunswick, and Trenton—each located in a different county and all characterized by high minority populations and elevated BMI risk. As part of that study, for SY 2019–20, school nurses in all public schools in the 4 study cities were asked to respond to a survey (either online via Qualtrics or on paper) to report the availability of foods and beverages offered on campus as well as the availability, delivery methods, and water quality in the school cafeteria during lunchtime in that SY. The school nurses were asked to consult with school food service personnel as needed to complete the survey. Surveys were sent to 110 schools in January 2020, and 96 responded before COVID-19-related school closures on 18 March 2020, for a response rate of 87%.

Water availability, delivery methods, and water quality were measured using questions from prior research [17]. To measure water availability, respondents were asked, “During [SY], has your school provided any of the following in the cafeteria during lunch time?” Items included the following: access to drinking water free of charge; free cups; and posted signs or other materials promoting water drinking. The response options were “Yes” and “No” to each item. The survey also asked about the delivery method and the number of different drinking water sources: “How many of each type of water source are available to students free of charge in the cafeteria during lunch time for the SY?” The types of water sources listed were as follows: drinking fountains; water dispensers (portable); refillable water stations; other (specify). Pictures of each water source were provided alongside this question to prevent any confusion regarding identification or categorization. Lastly, water quality was assessed with the question: “In your opinion, how often did the water source available to students in your cafeteria meet the following criteria for the current school year: clean (e.g., free from any visible dirt, gum, or trash); water temperature is cool; water pressure is good; water tastes good”. Response options included the following: most of the time, about half the time, rarely, don’t know/not applicable. Due to the small number of responses for “about half the time” and “rarely,” these two categories were combined for analysis.

Data on school level factors were obtained from the National Center for Education Statistics (NCES) and included total school enrollment, racial/ethnic composition of enrolled students (categorized as majority Hispanic, majority Non-Hispanic Black, majority Non-Hispanic White, or majority other), and the proportion of students eligible for free or reduced-price meals, divided into tertiles (first tertile represents schools with the fewest students eligible for free or reduced-priced meals while the third tertile represents schools with the highest percentage of students eligible for free or reduced-priced meals). In the US, school meals are federally funded through the US Department of Agriculture and are required to follow nutrition guidelines. Eligibility for free or reduced-price meals in the National School Lunch Program is based on household income where households with incomes at or below 130% of the federal poverty level (34,060 USD for a family of 4 in the 2019–20 school year) qualify for free meals, those between 131% and 185% (48,470 USD for a family of 4 in the 2019–20 school year) qualify for reduced-price meals, and those above 185% pay full price [23]. Analyses by school race/ethnicity were limited to schools classified as majority Hispanic and majority Non-Hispanic Black, as there were no schools identified as majority Non-Hispanic White or majority other. Lastly, schools were classified as either elementary or secondary (middle or high school) based on the grades offered. Due to the small sample size of middle schools, these were combined with high schools into a single “secondary schools” category, following previous studies [24,25]. 

### Statistical Analysis

All analyses were conducted using Stata 17.0 (StataCorp. 2021. Stata Statistical Software: Release 17. College Station, TX, USA: StataCorp LLC.). Statistical significance was set at *p* < 0.05, based on two-sided tests. Univariate statistics were used to calculate (1) the proportion of schools providing free drinking water and engaging in promotional efforts; (2) the mean number of water fountains, portable water dispensers, refillable water stations, and other drinking water sources per 100 students; (3) the frequency with which schools answered “most of the time” to water quality questions (clean, cool, good pressure, and good taste). Bivariate statistics were used to examine associations between school characteristics (school level, majority race, and FRPM eligibility tertile proportions) and key outcomes (water availability, water delivery method, and water quality). Group differences were evaluated using (a) t-tests for continuous outcomes (mean number of water sources per 100 students) and (b) Pearson’s chi-square tests for categorical outcomes (availability of free drinking water, availability of promotional material, and water quality). When the cell size was < 5, Fisher’s exact tests were used instead of Pearson’s chi-square tests. Missing survey responses among the returned surveys were addressed through multiple imputations [26]. Missing values ranged from 0% (whether free drinking water was offered) to 32% (whether the water tasted good) for the study variables.

## 3. Results

Table 1 reports the demographic characteristics of the participating schools. Most schools served a predominantly lower-income student population with high levels of eligibility for free and reduced-priced meals (average free and reduced-price eligibility rate was 76.8%), 56.3% were classified as majority Hispanic, while the remaining 43.7% were classified as majority Non-Hispanic Black. Nearly 70% of the schools in our sample were elementary schools.

Table 2 presents data on drinking water availability and quality among the 96 schools that responded to the survey. In SY 2019–20, 83 schools (86.5%) provided access to free drinking water in the cafeteria during lunch. Additionally, 47.9% of schools indicated (a) having promotional material for drinking water and (b) providing free cups for drinking water. The most common source of water was water fountains, available in 64 schools (66.7%), with a mean of 0.6 fountains per 100 students. Among these 64 schools, 20 (29.7%) had only water fountains as a water source, while the other 45 also had other sources of water. Among the 30 schools that reported having water coolers, the mean number of coolers was 0.6 per 100 students, with 9 schools relying solely on water coolers. Similarly, 30 schools had refill stations, with a mean of 0.4 refill stations per 100 students, with 2 schools having only refill stations as a source. Lastly, 39 schools reported having other water sources, with a mean of 1.0 per 100 students; for 6 of these 39 schools, the other water sources were water bottles. No school relied solely on “other” water sources. 

The overall water quality was rated positively, with similar responses across the four quality domains, with the majority of schools reporting water that was “clean most of the time” (79.2%), “cool most of the time” (78.1%), had “good water pressure most of the time” (78.1%), and “tasted good most of the time” (76.0%) (Table 3). When comparing water quality across the different types of water sources offered, there was no difference between the proportion of schools that answered “clean” or “cool” most of the time, but there were significant differences when comparing the proportion of schools that answered “good water pressure” and “tasted good” most of the time, with refill stations, water coolers, and fountains being rated highest.

There were no differences in water availability, water sources, or water quality by school level (Table 4). Statistically significant differences were observed in the proportion of schools with water fountains (*p*-value = 0.001), water coolers (*p*-value = 0.004), and refill stations (*p*-value = 0.016) across FRPM tertiles (Table 4). Specifically, schools in the tertile with the highest percentage of students eligible for FRPMs had the highest prevalence of water fountains (87.5%), while those with the lowest percentage of students eligible for FRPM reported the lowest (43.8%). Conversely, schools with the lowest percentage of students eligible for FRPM had the highest proportion of water coolers (53.1%). The prevalence of refill stations was 37.5%, 12.5%, and 43.8% for the lower, middle, and higher FRPM eligibility tertiles, respectively. There was no significant difference in the proportion of schools with “other” water sources across FRPM eligibility tertiles. Additionally, Table 4 also shows differences in the mean number of water sources per 100 students by school race/ethnicity, with majority Non-Hispanic Black schools having a higher number of each type of water source. There were no differences in water quality or water promotion measures by race/ethnicity or FRPM eligibility tertiles.

## 4. Discussion

In this study, we assessed the availability and quality of free drinking water in school cafeterias during lunch across 96 K–12 public schools from four New Jersey districts for SY 2019–20. Building on previous research on water availability and quality in schools nationwide [18] and in individual states such as California [16,17] and Massachusetts [19], our study also examined differences in water availability and quality based on school-level factors. 

While most schools in our sample provided free drinking water in the cafeteria during lunchtime, 13.5% did not, almost a decade after the passage of the HHFKA. These results align with those reported in previous studies, most based on reports from school personnel (food service staff or administrators). For example, in a nationally representative sample, 86.4% to 89.4% of schools offered free drinking water in SY 2009–11 (before the full implementation of the HHFKA) [18]. A similar level of water availability was reported in Massachusetts (76.3% of schools offered free drinking water) shortly after the passage of the HHFKA (SY 2011–12) [19]. A California study examining changes in free drinking water availability post-HHFKA found a 12% increase in water availability from the study period 2010–11 to study period 2016–18, with 87% of schools providing free drinking water post-HHFKA implementation [16]. Despite improvements in water availability post-HHFKA, collectively, these studies highlight gaps in water availability in schools.

The most common source of water in the cafeteria was water fountains, with 66.7% of schools in our sample reporting one or more water fountains in the cafeteria. This is lower than the proportion found in a nationally representative sample of US schools in SYs 2009–12, which reported water fountains in 74.5% of elementary schools, 69.8% of middle schools, and 72.1% of high schools [18]. Further, we found that schools with water fountains had 0.6 fountains (SD = 0.8) per 100 students on average, which is below the New Jersey plumbing code requirement of 1 fountain per 100 students and the 2015 National Standards Plumbing Code [N.J. Admin. Code § 5:23-3.15]. Thus, while 66.7% of surveyed schools report having water fountains, 0.6 fountains per 100 students would translate to 1 fountain per 167 students, which might limit students’ ability to truly access water at lunch. There is a possibility that this low fountain-to-student ratio is partially ameliorated via other sources of water, as nearly half of the study schools (n = 45) used other water sources, such as water coolers and refill stations, in combination with water fountains. On the other hand, nearly 21% of schools (20 out of 96) reported having only water fountains in their cafeterias. In those schools, water access may be more limited, given that student water consumption was reported to be 20% higher in schools that provided water in dispensers or bottleless water coolers compared to those with only traditional water fountains [27]. Further, students’ concerns about the safety and cleanliness of tap water in schools [21], and specifically the cleanliness of water fountains, could also limit their intake in schools that only have water fountains.

In addition, evidence suggests that water availability in schools might be lower than what is reported by school staff. For example, in a random sample of 24 California schools, only 58%, had free drinking water in the cafeteria based on direct observation [20]. Similarly, in 2011, just after the implementation of HHFKA, only 46% of 59 public middle schools in Minnesota were directly observed to provide free drinking water in the cafeteria compared to the 96% that was reported by the school food directors [16]. Our study was sent to school nurses who were instructed to consult food service staff regarding the availability and quality of water in the cafeteria; therefore, it is possible that our results are an overestimation of true water availability in these schools. If our findings are an underestimation, then it is likely that even fewer students have access to free drinking water in schools.

In addition to availability, water quality is a key predictor of students’ water consumption [14,15]. One in five elementary schools and more than a quarter of middle and high schools in the current study reported “no” to the free water offered in the cafeterias being “clean,” “cool,” “tasting good,” and/or “having good pressure.” These results align with previous research highlighting water quality challenges in schools. For instance, a study conducted in Massachusetts schools in May–June of 2012 reported that 70% of water sources were clean, while a survey of schools in California reported that 83% of schools in 2010–11 and 84% of schools from 2016–18 had clean water sources [16,19]. Further, when students were surveyed about the quality of drinking water available in their schools, 59% reported that their fountains were unclean, and 48% reported that their water did not taste good [17]. Lower perceived water quality from students may stem from different perspectives, as students may have a more critical view of the adequacy and quality of their drinking water compared to administrative school staff. Negative perceptions of the safety and cleanliness of water in schools, particularly tap water from drinking fountains, could also impact students’ water intake, as students might choose to avoid water from these sources and choose juice or milk instead. This issue is particularly relevant in states like NJ, which in response to water quality concerns in schools, became one of the first states to implement a mandatory lead testing program in all public schools in 2017. All NJ schools must (1) test their water every three years and, if elevated lead levels are found, (2) inform parents and (3) provide remediation plans [28]. However, despite these efforts, some schools in NJ continue to report lead levels above limits [29]. Even when remediation steps are taken, reports of lead contamination in schools can erode students’ and parents’ trust in school tap water, potentially reducing water intake.

The combined issues of limited availability and concerns about quality may play a role in both the water intake among school children as well as their intake of sugar-sweetened beverages. Emerging evidence supports this connection, when water consumption is made easier in the school setting, students drink more water and less soda [30]. In addition, several studies have documented improvements in BMI, especially for low-income children, after the implementation of the HHFKA [31,32], suggesting that improvements in school nutrition offerings, including beverages and water access, may be yielding measurable public health results.

While the majority of schools surveyed made free drinking water available to students, continued efforts are needed to meet the HHFKA requirements by expanding options beyond drinking fountains. Additionally, equal attention should be given to the quality of water sources, as studies have shown that students are less likely to drink water if they perceive the source as unclean [14,15]. To support these improvements, schools need both financial and technical assistance to assess their current water availability infrastructure and develop effective strategies for enhancing both access and quality of water in their schools. Further, systematic collection of data on water availability and quality through routine environmental assessments could provide useful insights into trends in water access. Finally, in addition to the availability and cleanliness of water sources, it is also important to consider other factors that impact students’ ability to access water in the cafeteria. Often, short lunch periods and limits placed on students’ ability to get up from their seats during lunch, both outside the control of the cafeteria staff, limit students’ ability to make use of the water that is available. Longer lunch periods and more flexibility regarding students’ ability to get out of their seats at lunch should be considered to ensure access to the drinking water that is available.

### Study Strengths and Limitations

One of the key strengths of the study is that it examined water access in schools almost a decade after the implementation of the HHFKA, which required all schools to provide free drinking water access in the cafeteria. The study was conducted in four large urban school districts that represented approximately 60,000 students. However, this study is not without limitations. First, the surveys were completed by school nurses with help from school food service personnel; prior studies have shown that school staff may overestimate water access when compared to direct observation by researchers [19,20]. It is possible that these results may have overestimated water availability. In addition, student input would have been a valuable datapoint in assessing the true accessibility of water, and future studies should attempt to include their perspectives whenever possible. All data were collected from schools in a single state, which may limit the generalizability of the findings, as water quality policies can vary across states despite the national HHFKA requirement for free water access during school meals. While the sample’s focus on lower-income, high-minority schools provides important insight into underrepresented populations, these findings may not reflect conditions in more socioeconomically or demographically diverse settings. Lastly, these results represent water availability at a single point in time and do not account for changes over time.

## 5. Conclusions

While the majority of the study schools provided access to clean, freely available water sources, students in over 13% of schools lack access to free drinking water during lunchtime, almost 10 years post-implementation of the HHFKA. In addition, the most common source of water is water fountains, which may limit true water availability due to students’ ability to access those fountains during meals limits on how much water a student can realistically drink with one trip to the fountain. Further, issues with water quality—specifically its cleanliness, temperature, and taste—persist in 20–30% of schools. Providing access to good-quality, free drinking water beyond traditional drinking fountains is essential for preventing dehydration and encouraging students to choose water over calorie-dense SSBs, which are associated with weight gain. Supporting schools in assessing their current water infrastructure and identifying opportunities to expand and improve access is an important step towards increasing water intake in schools and enhancing long-term student health.

## Figures and Tables

**Table 1:** Characteristics of the 96 study schools from New Jersey, for school year 2019–20.

	Mean (SD) or %
School student enrollment	618 (311)
Average % of students eligible for FRPM ^1^ by FRPM eligibility tertile	
Lower FRPM eligibility tertile (39.6–74.3%)	63.8 (9.4)
Middle FRPM eligibility tertile (74.4–83.8%)	79.1 (3.1)
Higher FRPM eligibility tertile (84.0–95.2%)	87.6 (2.9)
Majority race/ethnicity of enrolled students	
Hispanic	56.3%
Non-Hispanic Black	43.7%
School level	
Elementary school	68.8%
Middle/high school	31.2%
Total number of schools (N)	96

^1^ FRPM—free and reduced-price meal.

**Table 2:** Drinking water availability, method of delivery, and water quality in New Jersey schools, for school year 2019–20 (N = 96).

**Free Drinking Water Accessibility in Cafeteria During Lunch**	**Yes** **%**	**No** **%**
	86.5%	13.5%
**Water Sources Available in Cafeteria During Lunch**	**Yes** **%**	**Average per 100 Students ^1^** **Mean (SD)**
Schools with water fountains	66.7%	0.6 (0.8)
Schools with water coolers	31.3%	0.6 (0.8)
Schools with refill stations	31.3%	0.4 (0.5)
Schools with water sources other than above	40.6%	0.4 (0.4)
All water sources	-	1.0 (1.5)
**Quality of Drinking Water**	**Selected “Most of the Time”** **%**	**Selected “Half the Time”/“Rarely”** **%**
Clean (e.g., free from any visible dirt, gum, or trash)	79.2%	18.8%
Water temperature is cool	78.1%	22.9%
Water pressure is good	78.1%	22.9%
Water tastes good	76.0%	24.0%
**Water Promotion**	**Yes** **%**	**No** **%**
% of schools providing free cups for water during lunch	47.9%	50.3%
% of schools promoting drinking water	47.9%	50.3%

^1^ = calculated only among schools with the specified water source.

**Table 3:** Drinking water quality, availability of free cups, and promotion by water source in New Jersey schools, for the school year 2019–20.

Schools Reporting Drinking Water Quality Most of the Time as	Multiple Sources	Water Fountains Only	Refill Stations Only	Coolers Only	Other Sources Only	Total
Clean (e.g., free from any visible dirt, gum, or trash)	78.7%	95.0%	100%	80.0%	33.3%	79.2%
Water temperature is cool	75.4%	85.0%	100%	80.0%	33.3%	78.1%
Water pressure is good	70.5% *	95.0% *	100% *	90.0% *	33.3%	78.1%
Water tastes good	67.2% *	95.0% *	100% *	100% *	33.3% *	76.0%
Schools providing free cups for water during lunch	44.3% *	35.0%	100%	90.0% *	33.3% *	47.9%
Schools promoting drinking water	52.5%	40.0%	0%	60.0%	0%	47.9%
Total number of schools (N)	61	20	2	10	3	96

* = *p*-value of statistical significance (*p* < 0.05).

**Table 4:** Drinking water availability, method of delivery, and quality in New Jersey schools in comparison to demographic factors (school level, FRPM ^1^ eligibility tertile, and majority race), for school year 2019–20.

**Drinking Water Accessibility in Cafeteria During Lunch**	**n (%)**						
	**Elementary**	**Middle/high**	**Lower FRPM** **Eligibility**	**Middle FRPM** **Eligibility**	**Higher FRPM** **Eligibility**	**Hispanic**	**Non-Hispanic Black**
Schools with access to free drinking water	58 (87.9%)	25 (83.3%)	29 (90.6%)	24 (75.0%)	30 (93.8%)	45 (83.3%)	38 (90.5%)
Schools reporting no source of water	8 (12.1%)	5 (16.7%)	3 (9.4%)	8 (25.0%)	2 (6.25%)	9 (16.7%)	4 (9.5%)
**Water Sources Available in Cafeteria During Lunch**	**Mean (SD) or n (%)**						
	**Elementary**	**Middle/high**	**Lower FRPM** **Eligibility**	**Middle FRPM** **Eligibility**	**Higher FRPM** **Eligibility**	**Hispanic**	**Non-Hispanic Black**
Schools with water fountains, n (%)	45 (68.2%)	19 (63.3%)	**14 (43.8%)**	**22 (68.8%)**	**28 (87.5%)**	34 (63.0%)	30 (71.4%)
Average # of fountains/100 students ^2^	0.7 (0.9)	0.5 (0.4)	0.4 (0.3)	0.5 (0.3)	0.5 (0.6)	**0.4 (0.3)**	**0.8 (1.1)**
Schools with water coolers, n (%)	22 (33.3%)	8 (26.7%)	**17 (53.12%)**	**5 (15.6%)**	**8 (25.0%)**	15 (27.8%)	15 (35.7%)
Average # of coolers/100 students ^2^	0.6 (0.9)	0.6 (0.4)	0.5 (0.3)	0.5 (0.4)	0.7 (1.1)	**0.3 (0.2)**	**0.6 (0.6)**
Schools with refill stations, n (%)	20 (30.3%)	10 (33.3%)	**12 (37.5%)**	**4 (12.5%)**	**14 (43.8%)**	15 (27.8%)	15 (35.7%)
Average # of refill stations /100 students ^2^	0.5 (0.5)	0.4 (0.3)	0.6 (0.5)	0.3 (0.4)	0.9 (1.4)	0.4 (0.4)	0.8 (1.0)
Schools with water sources other than above, n (%)	29 (43.9%)	10 (33.3%)	9 (28.1%)	15 (46.9%)	15 (46.9%)	19 (35.2%)	20 (47.6%)
Average # of other water sources /100 students ^2^	0.4 (0.4)	0.3 (0.3)	0.3 (0.1)	0.3 (0.3)	0.5 (0.5)	**0.2 (0.1)**	**0.5 (0.4)**
Average number of all water sources per 100 students ^2^	1.1 (1.9)	0.8 (0.7)	0.8 (0.7)	0.8 (0.7)	1.4 (2.4)	**0.6 (0.6)**	**1.4 (2.1)**
**Quality of Drinking Water**	**%**						
	**Elementary**	**Middle/high**	**Lower FRPM** **Eligibility**	**Middle FRPM** **Eligibility**	**Higher FRPM** **Eligibility**	**Hispanic**	**Non-Hispanic Black**
Schools reporting drinking water quality most of the time as							
Clean (e.g., free from any visible dirt, gum, or trash)	81.8%	80.0%	75.0%	84.4%	84.4%	44 (81.5%)	34 (81.0%)
Water temperature is cool	80.3%	70.0%	75.0%	71.9%	84.4%	42 (77.8%)	32 (76.2%)
Water pressure is good	80.3%	70.0%	78.1%	68.8%	84.4%	41 (76.0%)	33 (78.6%)
Water tastes good	77.3%	73.3%	78.1%	81.3%	68.8%	39 (72.2%)	34 (81.0%)
**Water Promotion**	**%**						
	**Elementary**	**Middle/high**	**Lower FRPM** **Eligibility**	**Middle FRPM** **Eligibility**	**Higher FRPM** **Eligibility**	**Hispanic**	**Non-Hispanic Black**
% of schools providing free cups for water during lunch	32 (48.5%)	14 (46.7%)	20 (62.5%)	13 (40.6%)	13 (40.6%)	25 (46.3%)	21 (50.0%)
% of schools promoting drinking water	33 (50.0%)	13 (43.3%)	16 (50.0%)	18 (56.3%)	12 (37.5)	25 (46.3%)	21 (50.0%)
Total number of schools (N)	66 (100%)	30 (100%)	32 (100%)	32 (100%)	32 (100%)	54 (100%)	42 (100%)

^1^ FRPM—free and reduced-price meal; ^2^ = calculated only among schools with the specified water source; bolded text represents statistical significance at *p* < 0.05.

## Data Availability

The data presented in this study are available on request from the corresponding author, as these data are part of a much larger and more complex study. The full data set we used also contains a large amount of data that is not relevant to this particular study.

## Abbreviations

HHFKA	Healthy Hunger-Free Kids Act
FRPM	Free and Reduced-Priced Meals
NJCHS	New Jersey Child Health Study
NSLP	National School Lunch Program
SSBs	Sugar-Sweetened Beverages
SY	School Year
